# 3D structured illumination microscopy of mammalian embryos and spermatozoa

**DOI:** 10.1186/s12861-015-0092-7

**Published:** 2015-11-26

**Authors:** Jens Popken, Maik Dahlhoff, Tuna Guengoer, Eckhard Wolf, Valeri Zakhartchenko

**Affiliations:** Division of Anthropology and Human Genetics, Biocenter, LMU Munich, Grosshaderner Str. 2, D-82152 Planegg-Martinsried, Germany; Chair for Molecular Animal Breeding and Biotechnology, and Laboratory for Functional Genome Analysis (LAFUGA), Gene Center, LMU Munich, Feodor-Lynen-Str. 25, D-81377 Munich, Germany; Chair for Molecular Animal Breeding and Biotechnology, LMU Munich, Hackerstr. 27, D-85764 Oberschleissheim, Germany

**Keywords:** Super-resolution fluorescence microscopy, Mammalian embryos

## Abstract

**Background:**

Super-resolution fluorescence microscopy performed via 3D structured illumination microscopy (3D-SIM) is well established on flat, adherent cells. However, blastomeres of mammalian embryos are non-adherent, round and large. Scanning whole mount mammalian embryos with 3D-SIM is prone to failure due to the movement during scanning and the large distance to the cover glass.

**Results:**

Here we present a highly detailed protocol that allows performing 3D-SIM on blastomeres of mammalian embryos with an image quality comparable to scans in adherent cells. This protocol was successfully tested on mouse, rabbit and cattle embryos and on rabbit spermatozoa.

**Conclusions:**

Our protocol provides detailed instructions on embryo staining, blastomere isolation, blastomere attachment, embedding, correct oil predictions, scanning conditions, and oil correction choices after the first scan. Finally, the most common problems are documented and solutions are suggested. To our knowledge, this protocol presents for the first time a highly detailed and practical way to perform 3D-SIM on mammalian embryos and spermatozoa.

## Background

With an axial resolution of about 100 nm and a lateral resolution of about 300 nm 3D structured illumination microscopy (3D-SIM) features an 8-fold volumetric resolution improvement over confocal microscopy [1, 2]. This improvement is achieved by redirecting the laser beams through a mobile grid and scanning each section 15 times with a slightly altered grid location and orientation (3 angles with 5 phases each). The signals produced at the borders of this mobile grid allow a software to compute super-resolution images [3–6]. This scanning and calculating procedure has three requirements for specimens. Firstly, 15 scans per section require superior bleach resistant signal providing molecules. This is especially important when scanning thick objects since the total exposure time throughout all sections increases with the total number of z-sections scanned. Secondly, all signal providing molecules must remain at their 3D location during the entire scanning process, otherwise the calculation of the final image stack will fail. Since complete stacks are scanned for each angle before rotating the grid, even the slightest movement may reduce image quality markedly. Even individual signal providing molecules moving through the object such as particles can greatly interfere with the algorithm. Thirdly, 3D-SIM is a form of wide-field microscopy. This means that sharp images can only be acquired in an area at a certain distance to the cover glass. Signals outside the optimal range will appear less crisp and image artifacts such as artificial generation of signals can occur. This requires the use of the correct oil for a specific distance to the cover glass. Because of these three requirements flat and adherent cells are the optimal choice for 3D-SIM. However, blastomeres of mammalian embryos are non-adherent, large and surrounded by the zona pellucida. We have developed a protocol for 3D-SIM on mammalian embryos and generated high quality data for multiple studies [7, 8]. Here we present a detailed experimental protocol how to achieve superior image quality of mammalian blastomeres using 3D-SIM.

## Methods

### Ethics statement

All animal procedures and experiments with embryos were approved by the Government of Upper Bavaria (permit number 55.2-1-54-2532-34-09) and performed in accordance with the German Animal Welfare Act and European Union Normative for Care and Use of Experimental Animals.

### Recovery and culture of mouse embryos

Female mice were injected interperitoneally (IP) with pregnant mare’s serum (PMS) between 1:00 and 4:00 PM of Day 1. On Day 3, forty-two hours after the PMS injection, the mice received an IP injection of human chorionic gonadotropin (HCG). Immediately following injection, females of the FVB/N inbred strain were mated with males of the same mouse strain. Ovulation occurs approximately 12 h after HCG injection, at which time the eggs can be fertilized. Females were screened every morning and evening for vaginal plugs to see if mating has occurred and sacrificed by cervical dislocation at the same day after finding a vaginal plug (0.5 dpc). For embryo recovery, females were euthanized by cervical dislocation under isoflurane anesthesia. The oviduct was removed and flushed with M2 medium (Sigma, Taufkirchen, Germany) containing 0.4 % bovine serum albumin (BSA) (Roth, Karlsruhe, Germany). Zygotes were collected under a stereo microscope (Zeiss, Jena, Germany) with 20x magnification and transferred to microdrops of M16 medium (Sigma) on a culture dish covered with paraffin oil (Roth). Embryos were cultivated at 37 °C and 5 % CO_2_ in an incubator until the appropriate stage for fixation.

### Recovery and culture of rabbit embryos

Recovery of embryos was performed as described [9]. Female Zika rabbits were first superovulated by injection of 100 IU (international units) of equine chorionic gonadotropin (ECG; Intergonan, Intervet) intramuscularly and 100 IU of human chorionic gonadotropin (HCG; Ovogest, Intervet) intravenously 72 h later. 18-20 h post-HCG injection and after natural mating *in vivo* fertilized zygotes were flushed from the explanted oviducts of rabbits in warm phosphate buffered saline (PBS) supplemented with 4 mg/ml bovine serum albumin. Rabbit embryos were cultured in Quinn’s medium (SAGE, Trumbull, CT) containing 2.5 % fetal calf serum (FCS) in a humidified atmosphere of 5 % CO_2_ in air at 38.5 °C until the appropriate stage for fixation.

### Recovery and culture of bovine embryos

*In vitro* fertilization of bovine embryos was performed as described [10]. Cumulus-oocyte complexes (COCs) were obtained by aspiration from ovaries of slaughtered cows. COCs were matured in modified Parker’s medium containing TCM199 supplemented with 5 % estrous cow serum (ECS) and 0.2 U/ml o-FSH (Ovagen; ICPbio) for 20–22 h at 39 °C, 5 % CO_2_ in air and maximum humidity. Matured COCs were washed with the fertilization medium Tyrode's albumin lactate pyruvate (FERT-TALP) supplemented with sodium pyruvate (2.2 mg/ml), heparin sodium salt (2 mg/ml), and BSA (6 mg/ml) and transferred to 400-μl droplets of medium. Frozen spermatozoa were thawed at 38 °C. 100 μl thawed sperm suspension covered by 1 ml capacitation medium was subjected to the swim-up procedure for 60 min. The COCs and spermatozoa (2 x 10^6^ cells/ml) were co-incubated for 18 h at 39 °C and 5 % CO_2_ in humidified air. Presumptive zygotes were mechanically denuded by vortexing, washed 3x in SOF culture medium enriched with 5 % ECS, BME 100x (20 μl/ml; Invitrogen) and MEM (Minimum Essential Medium) 100x (10 μl/ml, Invitrogen), and transferred to 400-μl droplets of medium covered with mineral oil. Embryos were grown at 39 °C in a humidified atmosphere of 5 % CO_2_, 5 % O_2_, and 90 % N_2_ until they reached the appropriate stage for fixation.

### Cover glass preparation

Clean cover glasses were covered by polylysine (33 μl of a 1:100 Polylysine dilution in aqua bidest for 5 min) applying it in the exact center of each cover glass. If the covered area was too large the Vectashield applied to this prepared area at the final steps of the embedding procedure touched the constructed chamber’s walls and could leave the blastomeres uncovered and dried out (see below). When finished with the last cover glass, but at least 5 min after the start with the first one, the drop of polylysine solution was rinsed off the cover glasses in the same order with 500 μl-1000 μl aqua bidest. No drops were left, since after drying they could leave marks. The polylysinated side in the same corner of each cover glass was marked with a permanent marker and with a character or number that can only be read correctly from one side (for example "1"). Even though no liquid was visible on the slides they were dried for at least 2 h.

### Slide preparation

**Fig. 1:**
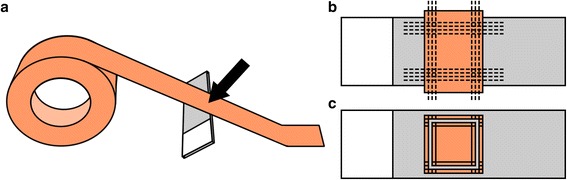
Preparation of slides. **a** The film was attached to the slide. The black arrow marks the spot where the film was pressed against the slide in a circular motion. **b** The film layers were cut with a scalpel. The dashed lines mark the cutting positions. **c** The slide was prepared for storage. The grey areas inside the outer border mark Tesa-Film removed with 'tweezers'

Clean glasses were covered with 4 layers of Tesa-Film. To cover a glass its center was pressed at a 45° angle against the sticky side of the film (Fig. 1a). Rotating a finger slowly in a circular pattern, the film was carefully pressed against the glass and attached onto the glass without generating bubbles. Then the film was cut off before and after the attached region on the glass. This procedure was repeated 3 more times to obtain 4 layers of Tesa-Film exactly on the top of each other on the glass. The reason for building such a high chamber lies in the effect of the objective pressing down and thereby reducing the distance between the cover glass and the slide inside the chamber. According to the equation of continuity A1*v1 = A2*v2 (A1, A2 = cross-sectional areas before and after distance reduction; v1, v2 = speed of the embedding medium before and after distance reduction) the flow speed of the embedding medium inside the chamber will increase when pressing against the cover glass with the objective. A larger initial volume in the chamber equals a smaller relative volume decrease which equals a smaller speed increase under the pressure of the objective. Furthermore, the slide was placed under a stereo microscope and using a scalpel and tweezers the pattern shown in Fig. 1b was cut into all 4 layers. The central area should be as large as possible to prevent contact between embedding medium and film when placing the cover glass on top of this structure. The parts shown in Fig. 1c were removed with tweezers except for the center part. This central area of the film remained attached during storage and was removed shortly before placing the cover glass on top of this construct in order to minimize dust within the central chamber.

### Fixation

Unless noted otherwise fixation of embryos and all subsequent steps were performed at room temperature (RT). For preservation of the 3D shape of embryos and nuclei it was necessary to avoid any deforming pressure and prevent embryos from drying out at any step of the following procedures. Embryos were briefly washed in 38 °C 1x phosphate buffered saline (PBS), fixed in 2 % paraformaldehyde (PFA) in PBS for 10 min, washed twice in PBS and then stored at 4 °C in PBS until further use. The maximum allowed storage duration depended on the concentration of target proteins for staining. Target proteins with a low concentration such as histones were stained and scanned shortly after fixation because the achievable signal strength diminishes with time. Target proteins with a high concentration such as nuclear lamins or nucleoporins were stained and scanned even after months of storage in PBS with acceptable signal strengths compared to the background signals.

### Immunostaining

Background caused by PFA was quenched using 20 mM glycine in PBS for 10 min. After washing twice with PBS embryos were permeabilized with 0.5 % Triton-X 100 for 15-30 min. After washing twice with PBS unspecific background signals were reduced by incubation in 2 % BSA for 2-4 h at RT. When targeting proteins with a low concentration, such as histones, embryos were blocked before staining for 4 h or longer in 2 % BSA in PBS to differentiate actual signals from background more efficiently. Four hours of blocking could result in a more focused localization of the signals leading to a slightly altered appearance compared to blocking for 2 h. When targeting proteins with a high concentration such as nuclear lamins or nucleoporins embryos were blocked before staining for only 2 h since the background was relatively less intense. However, maximum image quality was achieved with a blocking for 4 h. Then embryos were sequentially incubated in 40 μl of primary and secondary antibody solutions contained within PCR-tube caps, diluted in PBS with 2 % BSA. The source of the first antibody was different from the species of the embryo or the zona pellucida to avoid staining problems (see Troubleshooting below). In the case of using the same source as the embryo’s species, the zona pellucida was removed prior to staining as described below. Tables 1 and 2 show examples of primary and secondary antibodies used successfully with 3D-SIM. Specimens were incubated with primary antibodies overnight at 4 °C. After washing in PBS for 5 times with 2 % BSA the appropriate secondary antibodies, diluted in PBS with 2 % BSA, were applied for 1 h, again followed by washing in PBS for 5 times with 2 % BSA and 5 times without BSA. BSA had two effects. It blocked background signals and made embryos less sticky. While the first property was desired the second one was counter-effective when trying to attach blastomeres on a cover glass for minimal movement. In order to maximize stickiness even after 5 times of washing away BSA with PBS the following fixation step was used to bind the remaining BSA. After washing away the PFA embryos were extremely sticky and in order to prevent their attachment to any surface PBS was constantly pumped under the embryos to keep them floating within the PBS and transporting the embryos not within the transferpettor’s pipette but in a tiny drop at the tip of it. If the embryo has attached to a surface recovery was possible by blowing the embryo with the PBS very strongly against it. However, the pressure produced by a standard size opening of a pipette on a transferpettor may not suffice. In this case, the tip of a new glass pipette was melted until the opening was barely visible. Coating the pipette with silicone and the surfaces with silicone or agar may prevent embryos from attaching prior to the final embedding step. A cover glass with the polylysinated side upwards was put into the middle of the cover of a 3.5 cm well. A drop of 10-20 μl PBS was added in the center of the glass. Fixation of antibodies was performed with 4 % PFA in PBS for 10 min, followed by washing twice in PBS. PFA could cause increased background signals in the 488 nm channel. Therefore, fixing antibodies with only 2 % PFA in PBS was used to reduce such additional background signals. Chromatin was counterstained with DAPI (Life Technologies, Catalog No. D1306; 4′,6-diamidino-2-phenylindole) 25 μg/ml diluted in PBS for 15 min followed by washing twice in PBS.

**Table 1:** Examples of primary antibodies used successfully with 3D-SIM

Host	Target	Official name	Dilution	Company	ID
Goat	Epitope at C-terminus of Lamin B1. Detecting Lamin B1 and, to a lesser extent, Lamin B2	Lamin B Antibody (M-20)	1:100	Santa Cruz	SC-6217
Mouse	NUP153	Anti-NUP153 [QE5] antibody	1:200	Abcam	ab24700
Rabbit	H3K4me3	Anti-Histone H3 (tri methyl K4) antibody - ChIP Grade	1:100	Abcam	ab8580
Mouse	H3K9me3	Histone H3K9me3 antibody (mAb)	1:100	Active Motif	39285/6
Rat	RNAPII-S5p	Monoclonal antibody against CTD phosphoserine epitope 5	1:20	Gift from Dirk Eick (see Markaki et al. [23])	
Mouse	B23	Monoclonal Anti-B23 antibody produced in mouse	1:1000	Sigma	B0556

The host should be different from the species of the embryo. Otherwise, the zona pellucida should be removed prior to staining

**Table 2:** Examples of secondary antibodies used successfully with 3D-SIM

Host	Target	Fluorophore	Excitation [nm]	Dilution	Company	ID
Donkey	Mouse IgG (H + L)	Alexa	488	1:400	Invitrogen	A-21202
Donkey	Mouse IgG (H + L)	Alexa	594	1:500	Invitrogen	A-21203
Donkey	Goat IgG (H + L)	Alexa	488	1:400	Invitrogen	A-11055
Donkey	Goat IgG (H + L) preadsorbed	Alexa	594	1:400	Abcam	ab150136
Donkey	Rabbit IgG (H + L)	Dylight (Alexa bleaches less)	488	1:300	Jackson Immuno Research	711-485-152
Donkey	Rabbit IgG (H + L)	Dylight (Alexa bleaches less)	594	1:500	Jackson Immuno Research	711-505-152
Goat	Mouse IgG (H + L) preadsorbed	Alexa	594	1:300	Invitrogen	A-11032
Donkey	Rat IgG (H + L)	Alexa	594	1:300	Invitrogen	A-21209

### Isolation of blastomeres and attachment to the cover glass

**Fig. 2:**
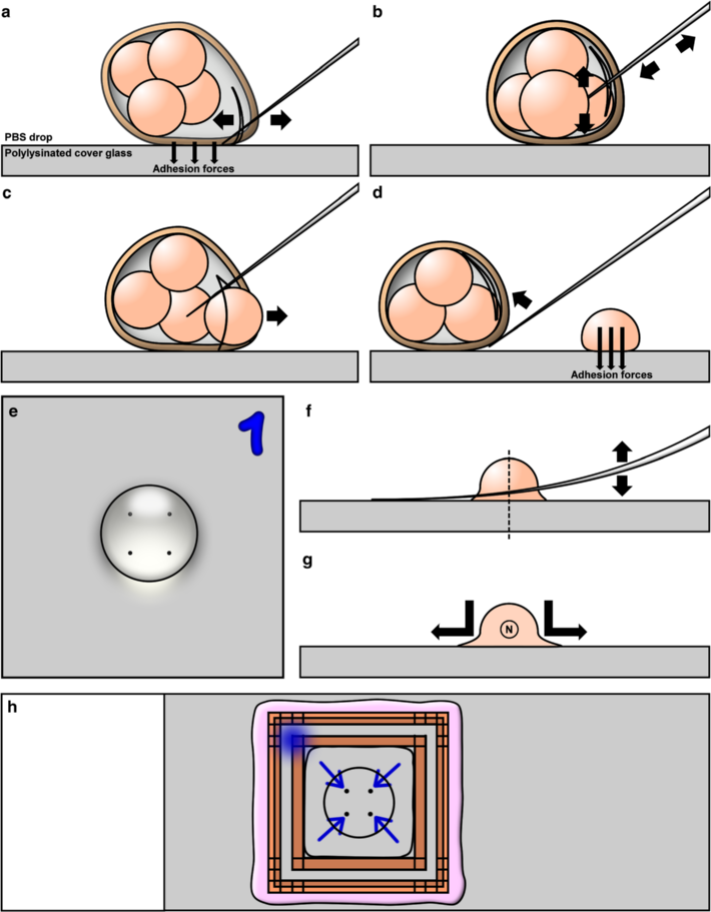
Isolation and attachment of individual blastomeres. **a** The zona pellucida was cut open. **b** A blastomere was isolated. **c** The isolated blastomere was moved outside the zona pellucida through the cut. **d** The zona pellucida with the remaining blastomeres was moved to the target location for the next blastomere. **e** Positions of blastomeres on the cover glass were chosen as far away from each other as possible, but not too close to the border of the PBS drop. **f** Once all blastomeres had been placed on the cover glass their outermost parts were pressed against the glass. The dashed line marks the section shown in G. **g** The attachment surface was increased even further by stretching out the pressed down periphery of blastomeres by moving the pressed down needle away from the blastomere. The circled N marks the position of the nucleus in this blastomere. **h** The finalized slide with the reversed cover glass attached in the center chamber and sealed with nail polish. The marker "1" was dissolved by the nail polish

**Fig. 3:**
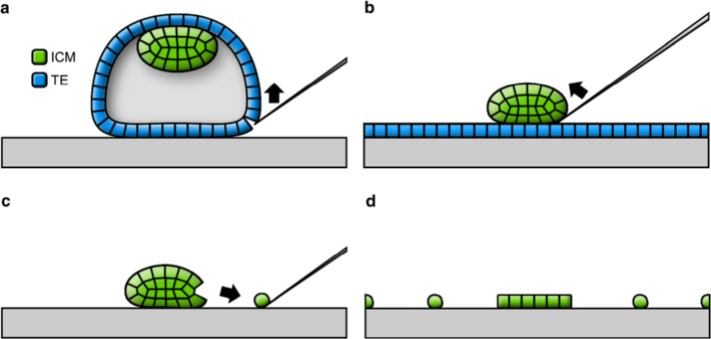
Isolation of blastomeres from blastocysts. **a** The trophectoderm (TE) layer was cut open. **b** The inner cell mass was moved away from the TE layer. **c** Blastomere isolation. **d** Individual blastomeres were placed remotely from each other

The 3.5 cm well containing the polylysinated cover glass, the drop of PBS and the embryo were transported to a microscope with at least one micromanipulator (two are preferred). An empty injection needle was mounted into the right micromanipulator holder. Similarly, a needle with a diameter smaller than an embryo was mounted into the left micromanipulator holder. This second needle was helpful to stabilize the position of the embryo but was not absolutely necessary for the subsequent steps. Once the embryo was placed inside the drop of PBS on top of the polylysinated cover glass adhesion forces attached the zona pellucida to the glass. An area of the zona pellucida slightly remote from the blastomeres was pressed against the cover glass with the injection needle. Then the needle was moved back and forth while keeping the zona pressed against the glass. The slit in the zona pellucida was enlarged by vibrating the needle via knocking against the micromanipulator with a finger (Fig. 2a). Afterwards the injection needle was inserted into the zona and stretched along the surface of the cover glass away from the embryo to open the zona even more. The embryo inside the open zona pellucida was rotated with the injection needle to line up the border between two blastomeres. Then the injection needle was placed along this border and moved back and forth to separate the blastomeres (Fig. 2b). Additional knocking against the micromanipulator could facilitate this process. A single blastomere was removed from the zona pellucida and the zona pellucida was moved with the remaining blastomeres away (Fig. 2c and d). The remaining blastomeres were moved inside the zona pellucida to a different remote location from the previous blastomere to prevent bleaching from scanning a neighbouring blastomere (Fig. 2e). The procedure was repeated for all blastomeres. The initial attachment of the blastomeres was automatically facilitated by the adhesion forces of the polylysinated surface. The attached blastomeres were left immobile otherwise this could lead to tearing and stretching artifacts. When working with blastocysts and the task was to scan only trophectoderm (TE) cells a blastocyst was let to sink on the glass and the monolayer of cells closest to the glass was scanned (Fig. 3a). The cell-free space inside blastocysts (blastocoel) can be large enough so that TE cells on the far side of the blastocyst do not interfere with the scans. If there were multiple layers of TE cells then individual TE cells were isolated and placed next to each other in remote locations as a monolayer. If inner cell mass (ICM) and TE cells were scanned the layer(s) of TE cells were cut like the zona pellucida. Tearing and stretching of cells remote from the slit were avoided. TE cells were then gently attached to the glass while the sphere of ICM cells was residing on top of this monolayer. This ICM sphere was then detached from the monolayer of TE cells by gently pressing against it with the side of the injection needle (Fig. 3b). The ICM sphere was then moved to a remote location and individual cells were isolated as described above for other embryos (Fig. 3c). To maximize image quality it was necessary to attach single blastomeres to the glass not touching each other (Fig. 3d). After attachment of all blastomeres to their target location additional attachments were performed to prevent movements disabling 3D-SIM reconstruction (Fig. 2f and g). The outermost part of blastomeres was pressed down with the injection needle against the cover glass. Then the 3.5 cm well was carefully rotated by 90° and the newly accessible outermost parts were pressed down. The zona pellucida was moved outside the drop so that it could not swim through the medium while scanning. When scanning sperm cells attached to the zona pellucida after an IVF procedure the zona was cut in smaller parts and these parts were pressed against the glass for maximum adhesion.

### Embedding

The closed well was transported back to the stereo microscope. The excess of PBS was removed from the cover glass while keeping all blastomeres covered with it. The cover glass was washed two times with 10-20 μl of PBS and covered slowly with 10-20 μl of Vectashield. The excess of Vectashield was removed from the cover glass while keeping all blastomeres covered with it. The slide with the removed central part of the Tesa-Film was placed under the stereo microscope. The cover glass with the drop facing down was put above the center part of the Tesa-Film frame and slowly lowered until the drop reached the slide avoiding the attachment of the drop with the Tesa-Film. The edges of the cover glass rested on the outer frame of the Tesa-Film. All sides of the outer frame were treated with a very small amount of nail polish using a fine brush. A contact between nail polish and Vectashield was avoided, while ensuring the complete sealing of the outer frame. After the nail polish had dried out the slide with the cover glass was held down at an angle under the stereo microscope and all blastomeres were marked with arrows on the back of the slide, but not on the cover glass (Fig. 2h). The slide was stored at 4 °C horizontally with the cover glass facing down to prevent movement of the Vectashield drop and detachment of the blastomeres.

### Oil selection for microscopy

Selection of the correct oil required to produce crisp images at the desired distance to the cover glass was performed in two steps. Firstly, the oil was estimated using embryos at various stages of development based on empirical testing (Table 3). We found that smaller blastomeres required oils with lower refractive indexes. Secondly, the correct oil was determined by analyzing preview and reconstructed images provided by 3D-SIM and its accompanying software package.

**Table 3:** Optimal refractive indices of oils per stage based on empirical testing

Stage/cell type	Optimal refractive index of oil
Zygote (centrally located pronuclei)	>1.534
2-cell	1.528–1.534
4-cell	1.526–1.528
8-cell	1.518–1.526
16-cell, morula and blastocyst	1.512–1.518
Spermatozoa, fibroblasts and cell lines	1.512–1.514

### Determination of the blastomere coordinates

A small drop of the estimated oil was applied on the clean cover glass. With the specimen localization microscope of the 3D-SIM setup the arrows in transmission light mode were found and the locations of the associated blastomeres were marked in the software.

### Determination of the correct oil before scanning

**Fig. 4:**
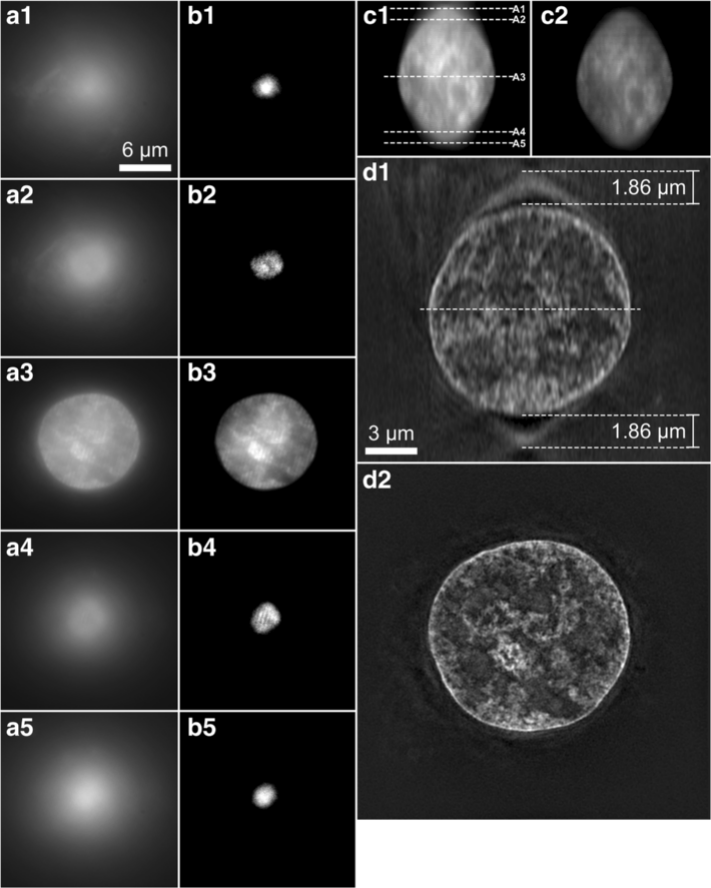
Determination of the correct oil before the first scan. **a1–5** Preview images of the first focal point of grid-free signals (**a1**), the first occurrence of the grid on the specimen (**a2**), the mid-section (**a3**), the last occurrence of the grid (**a4**) and the second focal point of grid-free signals. **b1–5** show the same images as in (**a1–5**) with optimized contrast for improved visualization of the grid. **c1** Orthogonal view of the first of the three non-reconstructed scans with different angles. The dashed lines mark sections (**a1**–**a5**). **c2** Orthogonal view of the second of three non-reconstructed scans. Note the reduction of signal strength between the two scans caused by bleaching. **d1** Orthogonal view of the reconstructed scan. The central dashed line marks the section of D2. **d2** Reconstructed mid-section with full resolution

The slide was transferred to the 3D-SIM microscope and the objective was moved in the z-direction at the position of the first blastomere. The z-position of the objective was compared before moving it towards the cover glass and afterwards. In our setup, smaller z-position values required oils with a higher refractive index, because the layer of the specimen was located at a greater distance from the cover glass. With this understanding, the preview image was used to predict the selection of the correct oil. In round specimens such as nuclei, wide-field microscopy apparently generates intense circular patterns that form cone shapes above and below the specimens (Fig. 4). The key to find the oil that provides a focused image in the vertical center of the specimen is to ensure that the distance of the upper and lower congregation points to the specimen is equal above and below the specimen (Fig. 4a–c). This can be assessed by moving in the z-direction above and below the specimen and counting sections between the top and bottom cone focal points and the first clearly visible grid on the nucleus’ top and bottom side. If the distance of the upper and lower congregation points was equal above and below the specimen then the focal section of the oil was in the specimen center. This was verified by checking for sharp lines of the grid in the mid-section. If the distance was not the same, then the oil with a higher refractive index was used in the case when the side of the nucleus with the lower z-position had a shorter distance between the cone focal point and the first clearly visible grid on the nucleus compared to the side of the nucleus with the higher z-position. If this scenario was reversed then the oil with a lower refractive index was used.

### 3D structured illumination microscopy (3D-SIM) and quantitative image evaluation

3D-SIM of embryonic nuclei was performed on a DeltaVision OMX V3 system (Applied Precision Imaging/GE Healthcare) with a lateral (x,y) resolution of ~120 nm and an axial (z) resolution of ~300 nm. The system was equipped with a 100x/1.40 NA PlanApo oil immersion objective (Olympus), Cascade II:512 EMCCD cameras (Photometrics) and 405, 488 and 593 nm diode lasers. Image stacks were acquired with a z-distance of 125 nm and with 15 raw SIM images per plane (5 phases, 3 angles). The SI raw data were computationally reconstructed with channel specific measured OTFs using the softWoRX 4.0 software package (Applied Precision). Images from the different color channels were registered with alignment parameters obtained from calibration measurements with 0.2 μm diameter TetraSpeck beads (Invitrogen). The voxel size of the reconstructed images is 39.5 nm in x,y and 125 nm in z with 32-bit depth. For all subsequent image processing and data analysis, images were converted to 16-bit composite tiff-stacks. Image stacks were processed using ImageJ 1.45b (http://rsb.info.nih.gov/ij/).

### Scanning

Before scanning the specimen was centered by comparing the distance of its border to the borders of the frame. This was also checked throughout all z-sections to use entire 3D-stacks. The key to great scans of perfectly immobile and clean specimens in clean Vectashield was to minimize bleaching during the scan. With highly concentrated target proteins such as lamins or nucleoporins a special property of the Alexa brand of secondary antibodies was used to not only reduce bleaching, but even stop or reverse bleaching. Alexa antibodies displayed two bleaching properties. Firstly, they bleached during a first intense scan (Table 4). Secondly, after such a first intense scan, their photon count was much more stable during a second less intense scan (Table 5). To obtain optimal results, Alexa stained specimens were scanned with a highly concentrated target protein in two steps. In the first step scanning was performed using the CCD camera with high laser intensity. This first reconstructed scan was inspected using the orthogonal viewer of the DV viewer software or with FIJI (ImageJ with appropriate plugins for viewing DV files) to check if the correct oil had been selected (Fig. 4). In that case, the two cones on the top and on the bottom of the sample had the same height. If the intensity of the signals did not allow for a second scan with the settings according to Table 5 then the first scan was repeated. The second scan was performed with a highly sensitive EMCCD camera with far less intense laser settings. A large photon count reduction was observed between the beginning and the end of the first, high intensity scan. The photon count stayed more stable in the second, low intensity scan. However, even the initial scan yielded very good results in many cases. This could be due to the fact that the bleaching of the fluorescent beads used to configure the algorithm of the software was proportional to the bleaching of the initial scan and therefore resembled the configured situation better than a reduced bleaching situation. Generally however, less bleaching was better than more bleaching. If the image quality was suboptimal when scanning multiple wavelengths at the same time then those were scanned in separate runs and the channels were combined after the scans using FIJI or the DV software provided with the microscope. The reduced image quality in parallel scans could be due to fluorophores being bleached by other wavelengths as well, adding additional bleaching during parallel scanning. For optimal results each channel was bleached independently until the scenario described in Table 5 was achieved for all channels at the beginning of the final scan. If the target was not highly concentrated multiple scans were avoided. In this case each channel was scanned independently with the second scan settings.

**Table 4:** Microscope settings and expected photon counts for the first scan

Fluorophor/staining	Mode	Exposure	Laser	Initial photon count
Alexa	CCD 5 MHz	25 ms	50 %	17000
DAPI	CCD 5 MHz	50 ms-120 ms (lower is better)	100 %	8000 - 16000

**Table 5:** Microscope settings and expected photon counts for the second scan

Fluorophor/staining	Camera	EMCCD gain	Exposure	Laser	Initial photon count
Alexa	EMCCD 5 MHz	2800-3300	30 ms	10 %	17000
DAPI	CCD 5 MHz	-	50 ms-120 ms (lower is better)	100 %	8000 – 16000

### Determination of the correct oil after the first scan

**Fig. 5:**
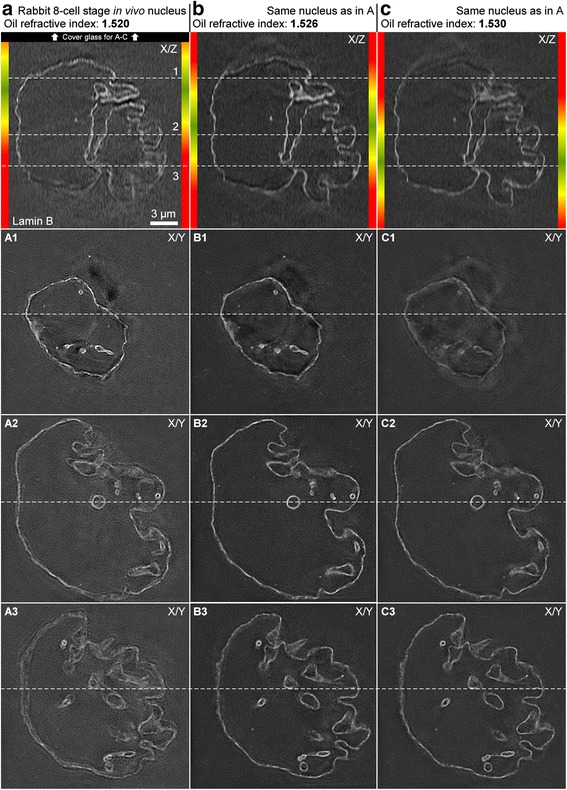
Effect of oils with different refractive indices. The same nucleus stained for lamin B from a rabbit 8-cell stage *in vivo* embryo scanned with 1.520 (**a**), 1.526 (**b**) and 1.530 (**c**) oils. Note the movement of the optimal focal section away from the cover glass with higher oils. The sides of the orthogonal views are color-coded for high quality sections at around the optimal focal section (green), medium quality sections further away (yellow) and low quality sections even further away (red)

Not all objects generated cones of artificially increased signals above and below the object. Non-round nucleus-like objects observed in early stage rabbit embryos did not generate intense cones (see the orthogonal views in Fig. 5). In this case, the correct oil was determined after the first scan. A highly concentrated target protein generating line-like signals along the z-axis like a nuclear lamina staining was the best indicator of whether to increase or lower the chosen oil refractive index and for this purpose orthogonal views of the first scan were generated. This was performed with the DV viewer software or with FIJI. Objects like nucleoli were identified in the stack and in the preview window to determine whether the top or the bottom of the z-stack were closer to the cover glass. To determine whether the top or the bottom of the scanned stack was closer to the cover glass the objective was moved up and down in the z-axis with the preview window and the z-position was compared with the objective’s position when it was at a large distance to the cover glass before scanning. The area of the z-stack was identified in the orthogonal viewer with the highest signal intensity and the sharpest lines (Fig. 5). This focus area was moved further away from the glass by changing to oil with a higher refractive index or moved closer to the glass by changing to oil with a lower refractive index. Once the correct oil had been identified all channels were scanned.

### Computing the final images

To ensure high quality scans the scanned stack was computed immediately after scanning. If the computed result was not optimal the same area was scanned again with different settings. In large scans the central area of 256 by 256 pixels was cut out and only this area was reconstructed for preview purposes. The final images were reconstructed with the optical transfer function (OTF) files generated using the optimal oil for fluorescent beads attached to the cover glass. In our case the refractive index of the oil optimal for beads was 1.512. Drift correction was activated to counteract any movements and produce images with a maximum quality. This setting did not have any negative effects on scans with perfectly still specimens.

### List of materials and reagents

**PMS:** pregnant mare’s serum

**HCG:** human chorionic gonadotropin

**Isoflurane**

**BSA:** bovine serum albumin

**M2:** medium

**M16:** medium

**ECG:** equine chorionic gonadotropin

**Quinn’s** medium

**FCS:** fetal calf serum

**Parker’s** medium

**TCM199:** medium

**ECS:** estrous cow serum

**FSH:** follicular stimulating hormone

**FERT-TALP:** fertilization medium Tyrode's albumin lactate pyruvate supplemented

**Heparin:** sodium salt

**SOF:** synthetic oviductal fluid

**BME:** Basal Medium Eagle

**MEM:** Minimum Essential Medium

**Polylysine**

**Vectashield**

**PFA:** paraformaldehyde

**Glycine**

**Triton-X 100**

**DAPI:** 4′,6-diamidino-2-phenylindole

## Results and Discussion

### Troubleshooting

**Fig. 6:**
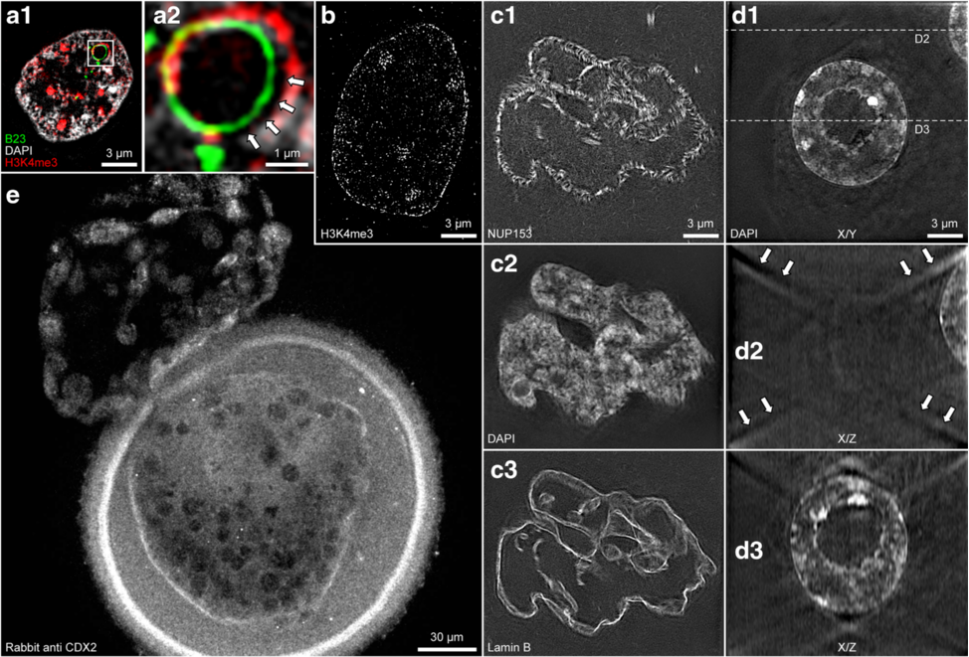
Potential problems. **a1**. Misaligned B23 (green), H3K4me3 (red) and DAPI (grey) channel. **a2** shows an enlargement of the box in **a1**. Arrows mark the gap between the signals that should be aligned. **b**. H3K4me3 staining at the periphery occasionally caused by using the blocking buffer developed by Markaki et al. [21] and utilized by Huebner et al. [22] without such an effect. **c1**. Movement during scanning may have caused short lines instead of round nuclear pores as visualized by a NUP153 staining. **c2**. The DAPI channel from the same nucleus as shown in **c2** is blurry. **c3**. The lines of lamin B of the same nucleus as shown in **c1** and **c2** are duplicated. **d1**. Blurry X/Y section of a DAPI stained nucleus next to another DAPI stained nucleus at the right top border. **d2**. The X/Z section marked in D1 shows lines emanating from all four corners caused by the second nucleus (marked by arrows; compare with Figure 4 D1). **d3**. These lines interfere with the signals produced by the central nucleus. **e**. Rabbit anti CDX2 staining of a rabbit blastocyst recorded by confocal microscopy shows a normal staining pattern outside the zona pellucida, a strongly stained zona pellucida and an altered staining pattern of blastomeres inside the zona pellucida when compared with nuclei outside the zona pellucida

In the case of a suboptimal image quality of scanned blastomeres we tested the staining and the microscope first using flat, adherent cells stained using the same conditions as stated above. If **channels were misaligned** (Fig. 6a) in adherent cells we recalibrated the microscope using fluorescent beads with the same temperature during scanning as for calibration. Misalignment could be checked best with prominent structures in multiple channels such as a lamin B and a nucleoporin staining or a nucleoli and a DAPI staining in nuclei with nucleoli lined by dense chromatin. If **antibodies for internuclear signals were also located at the nuclear periphery** (Fig. 6b) we used only 2 % BSA in PBS as blocking buffer since other additives could reduce accessibility of chromatin for antibodies. The antibody itself could also not be specific.

The wrong oil led to suboptimal results as described above. **Bleaching** was minimized by the pre-bleaching strategy outlined above. In the case of bleaching the top sections of a nucleus in an orthogonal view were brighter than the bottom sections. **Artificial short lines emanating from the real signals** (Fig. 6c) indicated sample movement during scanning. This could be either due to an improper attachment of the sample to the cover glass or movements of other materials inside the sample (loose fluorophores or particles). The solution was to re-attach the specimen and to fix the specimen again. To achieve this with the same sample, PBS was added to the hardened nail polish seal while gently disconnecting the bond between the nail polish and the cover glass with very thin tweezers. Once the nail polish seal and the Tesa-Film were disconnected from the cover glass on all sides the cover glass was lifted up using tweezers, turned in the way that the drop of Vectashield faced up and then placed inside a 3.5 cm well. All subsequent steps were performed by exchanging chemicals on the cover glass with the blastomeres still attached. Vectashield was washed away gently but completely with PBS for 5 times while not blowing against the attached blastomeres. The specimen was fixed with 4 % PFA in PBS for 10 min, washed twice with PBS and restained with DAPI 25 μg/ml diluted in PBS for 15 min followed by washing twice in PBS. The well was moved to a microscope featuring micromanipulators. The periphery of blastomeres was pressed onto the cover glass with an injection needle attached to a micromanipulator as described above. The well was moved back to a stereo microscope and the sample was washed gently without blowing against the blastomeres with PBS for 5 times. The different light direction options of the stereo microscope were used to check the medium for floating particles. All particles were removed with a pipette. The specimens were washed twice with Vectashield and checked again for particles. All particles were removed with a pipette. The cover glass was turned in the way that the Vectashield was facing down and then was slowly lowered to a fresh clean slide while keeping it horizontally aligned. The slide was sealed with nail polish and scanned again. Another factor for optimal image quality was to ensure that there were **no additional nuclei in close vicinity** (Fig. 6d) to the nucleus scanned. Nuclei in the vicinity could cause artificial patterns reducing the image quality. If the **zona pellucida displayed strong signals** (Fig. 6e) while blastomeres within the zona pellucida had low to no signals the secondary antibody was most likely targeting the embryo’s species. The zona pellucida could be saturated with target proteins. This is a common problem with mouse and rabbit embryos as most secondary antibodies are created in these species. The solution to this problem was to remove the zona pellucida before the first contact with antibodies. Handling sticky embryos after removal of 2 % BSA in PBS without a zona pellucida could be challenging. This required constant blowing under the embryos to prevent attachment to the bottom of the Eppendorf tube cap. When moving embryos between caps it was necessary to keep embryos in a drop of liquid at the tip of the transferpettor’s pipette to prevent attachment inside the pipette. In order to maximize stickiness even after 5 times of washing away BSA with PBS the following fixation step was used to bind the remaining BSA. After washing away the PFA embryos were extremely sticky and in order to prevent their attachment to any surface PBS was constantly pumped under the embryos to keep them floating within the PBS and transporting the embryos not within the transferpettor’s pipette but in a tiny drop at the tip of it.

To avoid the stickiness of embryos with or without the zona pellucida a more simple solution than a complicated pipetting system could be the use of chambers/slides/dishes covered by agar or silicon and pipettes covered with silicon. However, such additional reagents in direct contact with the solutions containing the embryos may create particles disturbing the scanning and calculating procedure. It should also be noted that the embryo’s stickiness is increased during prolonged storage of the fixed embryos at 4 °C, as prolonged storage can alter the surface of blastomeres and of the zona pellucida.

### Examples of 3D-SIM and confocal microscopy scans

**Fig. 7:**
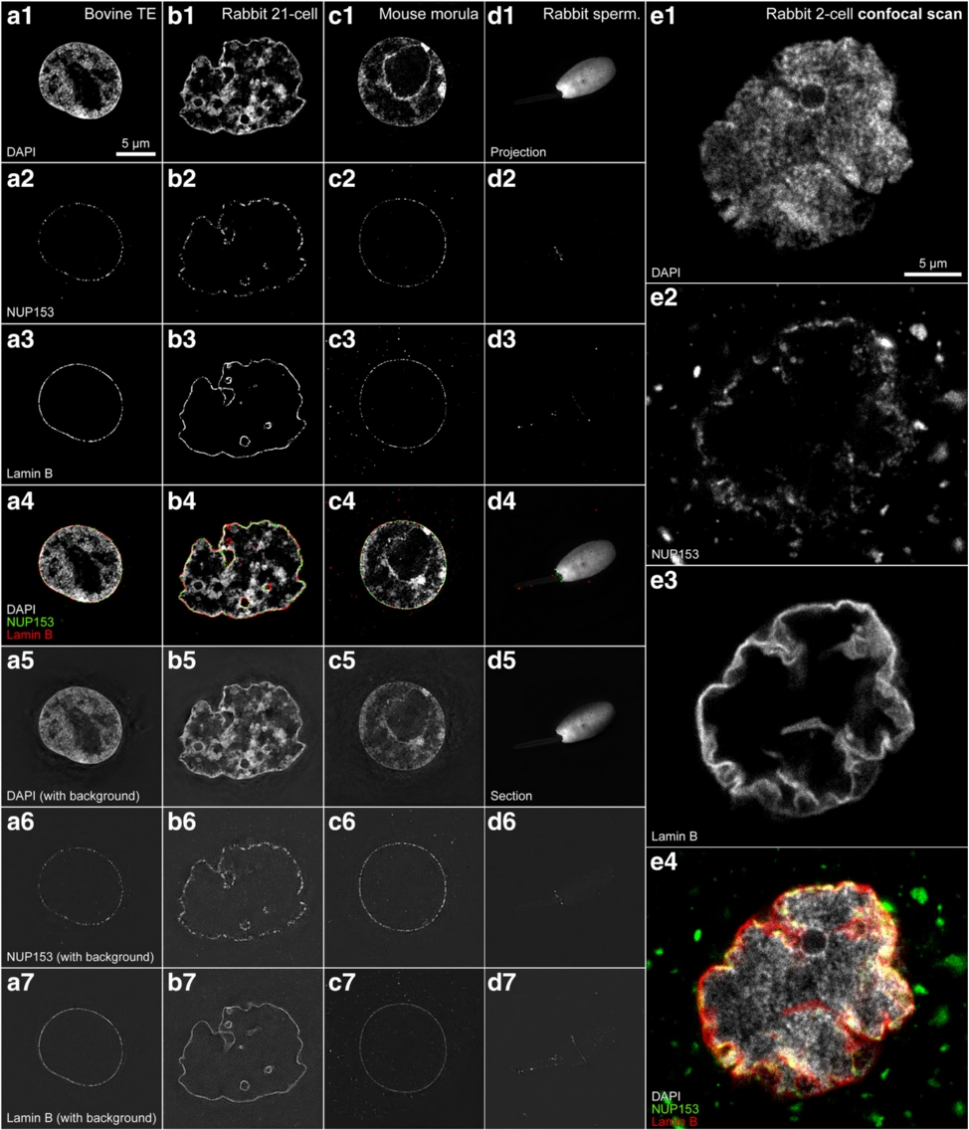
Examples of 3D-SIM and confocal microscopy scans. **a**. 3D-SIM scans of a nucleus in a trophectoderm blastomere from a bovine blastocyst. **b**. 3D-SIM scans of a nucleus in a blastomere from a rabbit 21-cell embryo. **c**. 3D-SIM scans of a nucleus in a blastomere from a mouse morula. The background may have increased slightly since this morula was stored for 2 years in PBS under mineral oil between fixation and staining. **d**. 3D-SIM scans of a rabbit spermatozoon. **e**. Confocal microscopy scans of a nucleus in a blastomere from a rabbit 2-cell stage embryo. **a1**–**e1**. DAPI. **a2**–**e2**. NUP153. **a3**–**e3**. Lamin B. **a4**–**e4**. Composite of DAPI (grey), NUP153 (green) and lamin B (red). **a5**–**d5**. DAPI without a correction of the background signals. **a6**–**d6**. NUP153 without a correction of the background signals. **a7**–**d7**. Lamin B without a correction of the background signals

Figure 7 features 3D-SIM scans of nuclei in blastomeres from cattle, rabbit and mouse embryos and rabbit spermatozoa. A confocal scan shows the difference in resolution.

This figure showcases the image quality possible with our 3D-SIM protocol by resolving individual nuclear pores.

### Comparison with alternative microscopy solutions

While 3D-SIM surpasses the image resolution of confocal imaging [11, 12], the stabilization of specimens, the determination of the correct oil, potentially increased bleaching by scanning each section far more often than during confocal microscopy and the requirement of reconstructing the final image make this method more demanding. Alternatively, confocal scanning without deconvolution may be the preferred method if signal intensities between multiple samples have to be compared, since algorithms can alter the signal intensity and create artifacts [13]. Wide-field microscopy also introduces new artifacts such as spherically increased signals to the specimens, which were not observed in confocal scans. GFP or RFP signals can get bleached too much with our setup and may require GFP or RFP boosters featuring Alexa fluorophores.

While the resolution of 3D-SIM is lower than the resolution achieved with electron microscopy [14, 15], 3D-SIM offers the advantage of using lasers with multiple wavelengths for multiple target co-localization studies not possible with electron microscopy. Additionally, embedding specimens in solid blocks and cutting/milling the blocks for scanning sections is necessary for electron microscopy and may introduce artifacts such as stretching of sections or cut marks [16, 17]. 3D-SIM can be performed in a liquid embedding medium that does not dry out and does not require cutting/milling and therefore does not change the morphology of specimens as demonstrated in completely spherical nuclei in 3D. However, liquid embedding media with a different density than PBS may potentially cause temporary deformation. A temporarily deformed zona pellucida was observed in earlier tests when embedding whole embryos with a zona pellucida in one step from PBS to Vectashield. Usually, the deformed zona pellucida recovered its original shape after some time. This temporary problem can be circumvented by washing embryos with increasing concentrations of the embedding medium in PBS before the final embedding step in pure embedding medium. Spherical nuclei in all dimensions prove that deformation based on medium density differences is either temporary or does not affect blastomeres. Earlier tests with embedding media that harden such as ProLong Gold led to alterations in morphology and an increased background signal and are therefore not recommended. This may be due to volume reductions during vaporization of the solvent.

### Applicability to alternative super-resolution fluorescence microscopy solutions

This protocol essentially aims to simulate the stability of fluorescent molecule localizations of adherent cells by attaching blastomeres to polylysinated cover glasses. Furthermore, it aims to simulate their proximity to the cover glass by using the correct oil to work around the distance to the cover glass. Therefore, because this protocol essentially turns blastomeres into simulated adherent cells close to the cover glass, it is feasible that this protocol is also compatible with other super-resolution microscopy solutions proven to work with adherent cells such as photoactivated localization microscopy (PALM) [18], stochastic optical reconstruction microscopy (STORM) [19] and stimulated-emission-depletion microscopy STED [20].

## Conclusions

To our knowledge, this protocol presents for the first time a highly detailed and practical way to perform super-resolution fluorescence microscopy on areas within blastomeres of mammalian embryos which can be remotely located from the cover glass. It underscores the importance of stabilizing the location of all signal providing molecules throughout the scanning process and demonstrates the process of achieving stability in non-adherent cells. Furthermore, this protocol gives practical advice in selecting the oil with the correct refractive index before the first scan is performed. Selection of the correct oil is mandatory for performing high quality wide-field microscopy at large distances from the cover glass such as nuclei in large, round blastomeres of early mammalian embryos.

While the image resolution surpasses confocal imaging, the stabilization of specimens, the determination of the correct oil, potentially increased bleaching by scanning each section more often than in confocal microscopy and the requirement of reconstructing the final image make this method more demanding. The problem of bleaching is far greater in large blastomeres that require scanning of many z-sections than in flat adherent cells. This protocol gives practical advice on how to utilize the non-linear bleaching properties of Alexa fluorophores to minimize bleaching in large stacks.

Finally, this protocol serves as a guide to generate super-resolution fluorescence scans of areas within blastomeres in a highly robust and reliable way.

## Abbreviations

3D: three-dimensional
B23: nucleolar phosphoprotein B23
BSA: Bovine serum albumin
CCD: Charge-coupled device
CDX2: Caudal-type homeobox 2
DAPI: 4′,6-diamidino-2-phenylindole
EMCCD: Electron multiplying charge coupled device
GFP: Green fluorescent protein
H3K4me3: Histone 3 lysine 4 trimethylated
ICM: Inner cell mass
IVF: *in vitro* fertilization
NUP153: Nucleoporin 153 kDa
PBS: Phosphate buffered saline
PFA: Paraformaldehyde
RFP: Red fluorescent protein
RT: Room temperature
SIM: Structured illumination microscopy
TE: Trophectoderm
